# High Yield of Adult Oligodendrocyte Lineage Cells Obtained from Meningeal Biopsy

**DOI:** 10.3389/fphar.2017.00703

**Published:** 2017-10-12

**Authors:** Sissi Dolci, Annachiara Pino, Valeria Berton, Pau Gonzalez, Alice Braga, Marta Fumagalli, Elisabetta Bonfanti, Giorgio Malpeli, Francesca Pari, Stefania Zorzin, Clelia Amoroso, Denny Moscon, Francisco J. Rodriguez, Guido Fumagalli, Francesco Bifari, Ilaria Decimo

**Affiliations:** ^1^Section of Pharmacology, Department of Diagnostics and Public Health, University of Verona, Verona, Italy; ^2^Group of Molecular Neurology, Hospital Nacional de Parapléjicos, Toledo, Spain; ^3^Laboratory of Molecular and Cellular Pharmacology of Purinergic Transmission, Department of Pharmacological and Biomolecular Sciences, University of Milan, Milan, Italy; ^4^Section of General and Pancreatic Surgery, Department of Surgery, Dentistry, Paediatrics and Gynaecology, University of Verona, Verona, Italy; ^5^Laboratory of Cell Metabolism and Regenerative Medicine, Department of Medical Biotechnology and Translational Medicine, University of Milan, Milan, Italy

**Keywords:** oligodendrocyte precursor cells, meninges, meningeal neural stem cells, myelin, oligodendrocyte differentiation, adult neural stem cells, spinal cord

## Abstract

Oligodendrocyte loss can lead to cognitive and motor deficits. Current remyelinating therapeutic strategies imply either modulation of endogenous oligodendrocyte precursors or transplantation of *in vitro* expanded oligodendrocytes. Cell therapy, however, still lacks identification of an adequate source of oligodendrocyte present in adulthood and able to efficiently produce transplantable cells. Recently, a neural stem cell-like population has been identified in meninges. We developed a protocol to obtain high yield of oligodendrocyte lineage cells from one single biopsy of adult rat meningeal tissue. From 1 cm^2^ of adult rat spinal cord meninges, we efficiently expanded a homogenous culture of 10 millions of meningeal-derived oligodendrocyte lineage cells in a short period of time (approximately 4 weeks). Meningeal-derived oligodendrocyte lineage cells show typical mature oligodendrocyte morphology and express specific oligodendrocyte markers, such as galactosylceramidase and myelin basic protein. Moreover, when transplanted in a chemically demyelinated spinal cord model, meningeal-derived oligodendrocyte lineage cells display *in vivo*-remyelinating potential. This oligodendrocyte lineage cell population derives from an accessible and adult source, being therefore a promising candidate for autologous cell therapy of demyelinating diseases. In addition, the described method to differentiate meningeal-derived neural stem cells into oligodendrocyte lineage cells may represent a valid *in vitro* model to dissect oligodendrocyte differentiation and to screen for drugs capable to promote oligodendrocyte regeneration.

## Introduction

Loss of oligodendrocytes in the CNS impairs neuronal transmission and increases neuronal frailty, eventually leading to cognitive and motor deficits (Karoutzou et al., 2007; Schmahmann et al., 2008; Duncan and Radcliff, 2016). The white matter of the adult CNS hosts a population of OPCs capable of generating myelinating oligodendrocytes in physiological conditions (Franklin and Ffrench-Constant, 2008; Rivers et al., 2008; Nishiyama et al., 2009). OPCs retain a degree of remyelinating ability in disease (Decimo et al., 2012a): in response to demyelinating insults, OPCs are activated, increase their proliferation and migrate to demyelinated sites, where they start to restore myelin coverage (Picard-Riera et al., 2002; Powers et al., 2013). However, in case of persisting and excessive demyelinating pathological microenvironment, such as in MS, ischemic, and traumatic injuries, OPCs are progressively depleted and their remyelination efficiency decrease (Guest et al., 2005; Franklin and Ffrench-Constant, 2008; Shi et al., 2015; Traka et al., 2016).

Restoration of adequate oligodendrocyte cell number and function can be envisaged either by manipulation and stimulation of the endogenous OPC pool or by transplantation of oligodendrocytes (Franklin and Ffrench-Constant, 2008; Gallo and Deneen, 2014; Najm et al., 2015; Steinbeck and Studer, 2015). Currently, there are no successful therapies available to promote remyelination (Franklin and Ffrench-Constant, 2008), and several limitations prevent oligodendrocytes transplantation (Franklin, 2015; Trounson and McDonald, 2015; Goldman, 2016). Ideally indeed, oligodendrocytes source should have the following properties: (i) be of adult origin; (ii) be accessible for sampling; (iii) be easily expanded *in vitro* and (iv) be collected and transplanted, after cell expansion, in the same patient (named autologous setting) without causing major adverse effects. Different cell populations have been evaluated for regenerative purposes, including OPCs (Nishiyama et al., 1999), ESCs (Trounson and McDonald, 2015), iPSCs (Ben-David and Benvenisty, 2011), and olfactory-ensheathing cells (OECs) (Murrell et al., 2008). Endogenous OPCs have been identified as NG2-expressing cells in the adult CNS; however, they are scattered throughout in the brain and spinal cord parenchyma (Nishiyama et al., 1999). Therefore, NG2-derived OPC extraction from the patient own reservoir is inapplicable due to the extended tissue sample required to obtain a sufficient number of cells (Nishiyama et al., 1999; Franklin and Ffrench-Constant, 2008; Schmahmann et al., 2008). On the other hand, ESCs are a potential unlimited source of oligodendrocytes. Ethical issues, however, raised by isolation from embryonic tissue together with the requirement of life-long immunosuppressive therapy for the transplant recipient, significantly compromise their clinical application (Trounson and McDonald, 2015). iPSCs are of adult origin and can efficiently differentiate into oligodendrocytes (Douvaras and Fossati, 2015) in large numbers; however, their clinical translation is dampened by their high risk of tumorigenicity (Ben-David and Benvenisty, 2011). Adult remyelinating cells from OECs represent a safer alternative (Fouad et al., 2005), as they can be expanded *in vitro* and transplanted in autologous settings (Murrell et al., 2008). Clinical trials using these cell sources showed promising results in terms of safety of cells grafting (Chen et al., 2014). Nevertheless, the presence and degree of remyelination obtained using these cell sources have not been described yet (Mackay-Sim et al., 2008). Overall, the identification of a cell source combining all these four properties (adult origin, accessible sampling, high yield of oligodendrocytes, and transplantable in an autologous setting) and that may represent a useful tool for high-throughput drug-screening assays for the identification of novel pharmacological targets for demyelinating disease is still under investigation (Franklin and Ffrench-Constant, 2008; Pino et al., 2017).

We described the presence of a pool of NSCs in rodent meninges (Bifari et al., 2009, 2015, 2017; Decimo et al., 2011, 2012a,b). Meningeal-resident NSCs display *in vivo* and *in vitro* gene expression properties similar to subventricular NSCs (Decimo et al., 2011; Bifari et al., 2017) and are able to migrate and differentiate into functional neurons in the neonatal cerebral cortex (Bifari et al., 2017). We described that cells with NSC features are present in meninges from the embryonic period up to adulthood (Bifari et al., 2009, 2015). Meningeal-resident NSCs can be cultured *in vitro* as neurospheres and differentiated into electrically functional neurons and oligodendrocytes (Bifari et al., 2009; Decimo et al., 2011). Considering the superficial localization of meninges on the CNS surface, adult meningeal-derived NSCs raise particular interest for their potential application in autologous cell transplantation and *in vitro* drug screening for demyelinating diseases. In this study, we developed a protocol to obtain high yield of remyelinating oligodendrocyte lineage cells from adult rat meningeal biopsy.

## Materials and Methods

### Organotypic Cell Culture

Animal housing and all experimental procedures were approved by the Istituto Superiore di Sanità (I.S.S., National Institute of Health; protocol N. 154/2014-B, Italy) and the Animal Ethics Committee (C.I.R.S.A.L., Centro Interdipartimentale di Servizio alla Ricerca Sperimentale) of the University of Verona (Italy). Six to eight weeks old male and female Sprague–Dawley rats were anesthetized by intraperitoneal injection with chloral hydrate (350 mg/kg) and sacrificed by cervical dislocation. Spinal cord meninges were collected under a stereomicroscope and small samples of approximately 1 cm^2^ were isolated; then, tissue samples were washed in ice-cold HBSS and cultured into 6-wells plates in neurosphere expansion medium (NS, see section Media Compositions). Every 3–4 days, half of the medium (approximately 3 ml) was substituted with fresh NS medium. After 7–10 days, neurospheres were collected, centrifuged, mechanically dissociated to a single-cell suspension and further expanded in NS medium or cultured in oligodendrocyte-inducing Step Go1 medium (see below).

### Media Compositions

#### NS Medium

Neurobasal medium (Thermo Fisher Scientific), 2% B27 supplement (Thermo Fisher Scientific), 1% N2 supplement (Thermo Fisher Scientific), 2 mM glutamine (Thermo Fisher Scientific), 100 U/ml penicillin and 100 μg/ml streptomycin (Thermo Fisher Scientific), 20 ng/ml human EGF (PeproTech) and 20 ng/ml FGF2 (PeproTech).

#### Step Go1 Medium

Neurobasal medium, 2% B27 supplement, 2 mM glutamine, 100 U/ml penicillin and 100 μg/ml streptomycin, 20 ng/ml human FGF2 and 20 ng/ml human PDGF-AA (PeproTech).

#### Step Go2 Medium

Neurobasal medium, 2% B27 supplement, 2 mM glutamine, 100 U/ml penicillin and 100 μg/ml streptomycin, 20 ng/ml human FGF2, 5 ng/ml human PDGF-AA and 15 nM 3,3′,5-triiodo-L-thyronine (T3) (Sigma–Aldrich).

#### Step Go3 Medium

Neurobasal medium, 2% B27 supplement, 2 mM glutamine, 100 U/ml penicillin and 100 μg/ml streptomycin, 5 ng/ml human PDGF-AA and 15 nM T3.

### Oligodendrocyte Differentiation

Single-cell suspensions, obtained from dissociated neurospheres, were cultured in Step Go1 medium to induce the differentiation into oligodendrocytes. After 7–10 days of culture, oligospheres are formed. Oligospheres were then dissociated into a single cell suspension and subsequently plated onto poly-D-lysine coated flasks for further 7–10 days in Step Go2 medium. After this period, Step Go3 medium was added for 3 days to prompt the differentiation process. At each of the four steps of oligodendrocyte differentiation we collected ∼100 k cells for immunofluorescence and ∼500 k cells for RT-PCR analyses.

### Immunofluorescence

Cells were plated onto poly-D-lysine coated glass slides. Staining procedure was performed as previously described (Bifari et al., 2015; Lange et al., 2016). Briefly, following fixation in 4% paraformaldehyde (PFA, Sigma–Aldrich), aspecific binding sites were blocked by incubation in blocking solution (3% fetal bovine serum, 1% bovine serum albumin, 0.3% Triton X-100 in PBS). Cells were incubated in primary antibody solution for 1.5 h at room temperature, washed thrice with blocking solution and incubated in the proper secondary antibody solution for 1 h. After three washes in blocking solution, slides were incubated for 10 min with the nuclear dye TO-PRO3 (Thermo Fisher Scientific) and mounted on glass microscope slides for confocal microscope quantification (Zeiss LSM 710 confocal microscope).

Primary antibodies: nestin (mouse, 1:1000, BD Pharmingen, cat# 556309), NG2 (rabbit, 1:1000, Merck-Millipore, cat# AB5320), oligodendrocyte marker O4 (O4) (mouse, 1:200, Merck-Millipore, cat# MAB345), oligodendrocyte marker O4 (O4) (mouse, 1:40, Sigma–Aldrich, cat# O7139), MBP (rabbit, 1:500, Dako, cat# A0623), MBP (mouse, 1:500, Sigma–Aldrich, cat# AMAB91064), GalC (rabbit, 1:100, Merck-Millipore, cat# AB142), NF160 (mouse, 1:200, Sigma–Aldrich, cat# N5264).

### Image Analyses and Quantification

To evaluate the oligodendroglial differentiation at each phase of the protocol, we performed immunofluorescence as described above. The positive-immunoreactive cells, for each marker, were determined using the ImageJ software (U.S. National Institutes of Health) as follow: (i) nestin positivity: cytoplasmatic elongated signal with single channel RGB color intensity ≥32 (with minimum value 0 = black; maximum value 255 = full color); (ii) NG2 positivity: cytoplasmatic elongated signal with single channel RGB color intensity ≥30 (with minimum value 0 = black; maximum value 255 = full color); (iii) O4 positivity: pointy signal in correspondence to the glycoprotein on membrane surface; (iv) MBP positivity: cytoplasmatic signal in cells with ≥3 positive branches; (v) GalC positivity: cytoplasmatic signal with single channel RGB color intensity ≥50 (with minimum value 0 = black; maximum value 255 = full color) and with ≥3 positive branches. We quantified a minimum of 50 cells for each differentiation stage for each marker (*n* ≥ 3 independent samples). Data were expressed as percentage of positive cells/total number of counted (TO-PRO3^+^) cells.

Cellular branches were manually counted for a minimum of 50 cells/sample/differentiation phase in blind quantification by three independent observers. Evaluation was performed on cells immunoreactive for MBP or GalC staining, which allowed visible identification of cellular branches, using the ImageJ software (U.S. National Institutes of Health). Data were expressed as mean number of branches/cell.

### NG2-Derived Oligodendrocyte Culture

Primary NG2-derived oligodendrocytes were isolated from mixed glial cultures prepared from postnatal day (P) 2 Sprague–Dawley rat cortex by shaking cells on an orbital shaker at 200 rpm, as previously described (Fumagalli et al., 2011, 2015). NG2-derived OPCs were then collected and separated from microglia by incubation for 20 min on an uncoated petri dish. Purified NG2-derived OPCs were seeded onto poly-D,L-ornithine-coated glass coverslips (50 μg/ml, Sigma–Aldrich) in 60 mm-dishes (300 k cells/dish) in Neurobasal with 2% B27, 2 mM L-glutamine (EuroClone), 10 ng/ml human PDGF-BB (Sigma–Aldrich), and 10 ng/ml human FGF2 (Space Import Export), to promote proliferation. Cells were maintained in proliferation medium for 4 days and, then, shifted to a differentiating medium containing 10 ng/ml T3 (Sigma–Aldrich) for 72 h. NG2-derived oligodendrocytes were lysed in 800 μl of TRIzol (Thermo Fisher Scientific).

### Quantitative Real-time RT (Reverse Transcription)-PCR Analysis (qRT-PCR)

Cells were collected at each stage of the differentiation protocol for *n* ≥ 3 replicates for *n* ≥ 2 independent experiments. qRT-PCR was performed as previously described (Bifari et al., 2009) using the following primers (forward and reverse) or Taqman^TM^ assays (Thermo Fisher Scientific):

*Nestin*: F-TTCTGGACCCCAAGCTGAAG;
R-GGGAGCACAGATCCCAGGTA
*Olig1*: F-TTACAGGCAGCCACCCATCT;
R-GAGCGGAGCTTCAGGCTTCT
*Cnp*: F-AGGCGTGCTGCACTGTACAA;
R-CCACATCCTGTTGGGCATATT
*Mog*: F-ATTGCCCTTGTGCCTATGCT;
R-TGCACGGAGTTTTCCTCTCA
*Mag*: F-CCAGCAGAGGACGGCATCTA;
R-GGGCTTCCAAGGTGCATACA
*Plp1*: F-TGCGCTGATGCCAGAATGTA;
R-TTGGAACTCGGCTGTTTTGC
*Dcx*: F-TTGCTTGTGGCCCTGAAAAG;
R-CCAGCTGTGGCAGATGGATT
*Tub3*: F-CCAAGTTCTGGGAGGTCATCA;
R-CCGAGTCCCCCACATAGTTG
*Syt1*: F-ACCAGCTGTTGGTGGGAATC;
R-ATCGGATGTACCCCCCATGT
*Aqp4*: F-CACCACGGTTCATGGAAACC;
R-AATCACAGCTGGCAAAAATGG
*Gfap*: Probe code Rn00566603_m1 (TaqMan^TM^ Assays)

The mRNA levels of the housekeeping gene β-actin were used as reference to normalize the expression of the genes of interest.

Data are expressed as relative gene expression levels compared to undifferentiated meningeal-derived stem cells (NS).

### Meningeal-Derived Oligodendrocyte Lineage Cells Transplantation in Focal Demyelination Rat Model

Animal housing and all experimental procedures were approved by the Bioethics Committee of The National Hospital of Paraplegics (Toledo, Spain). Three months old female Wistar rats, ∼300 g of weight (*n* = 6 animals for the LPC-control group, *n* = 6 animals for the LPC-transplanted group) were used. Rats were anesthetized with intraperitoneal injections of pentobarbital (40 mg/kg) and xylazine (10 mg/kg), the spinal cords were exposed by laminectomy at level of the T8 vertebra, and LPC (1% in saline solution) was injected at three points separated by 1 mm in the dorsal columns (2 μl at each point; 1 μl at each 0.7 and 0.5 mm of depth). In each injection point, the solution was administered at a rate of 0.5 μl/min by using a 33G needle and a 10 μl Hamilton syringe attached to a microinjector and a stereotaxic apparatus. The post-operative cares included subcutaneous injection of buprenorphine at 24 hours post injection (HPI) (0.03 mg/kg) and enrofloxacin (2.5 mg/kg) once daily until 5 days post injection (DPI). Moreover, animals received subcutaneous injections of saline solution for the first 5 DPI in decreasing doses, from 5 ml at 24 HPI to 1 ml at 5 DPI.

In order to perform meningeal-derived oligodendrocyte lineage cells transplantation, LPC-demyelinated animals were divided in two groups, LPC-control (not transplanted) and LPC-transplanted (transplanted with meningeal-derived oligodendrocyte lineage cells). Meningeal-derived oligodendrocyte lineage cells, at Step Go2, were transduced with an eGFP-expressing lentiviral vector [10 multiplicity of infection (MOI)] for 16 h. Spinal cords were again exposed at the T8 spinal level at 7 DPI, in order to inject vehicle (Neurobasal medium, LPC-control) or eGFP meningeal-derived oligodendrocyte lineage cells (LPC-transplanted). LPC-transplanted animals were injected with 2 μl of Neurobasal medium containing 100’000 eGFP cells/μl, while LPC-control group received an injection of 2 μl of Neurobasal medium. Injections were carried out at a rate of 0.5 μl/min using a 33G needle and a 10 μl NanoFil syringe attached to a microinjector and a stereotaxic apparatus. In each injection point, the needle was maintained for five further minutes to minimize the reflux of the solution. The bladders were emptied twice daily until cardiac perfusion for histological analysis.

### Luxol Fast Blue Staining Protocol

Myelin content was assessed in the tissue sections via LFB staining. Spinal cords from healthy control, LPC-control and LPC-transplanted animals were extracted after intracardiac perfusion with 4% PFA/4% sucrose and 25 μm-thick sections were cryosectioned. Sections from 1 cm-rostral to 1 cm-caudal to the lesion area were selected and used for analysis. First, 0.1% LFB solution was prepared solubilizing LFB (Sigma–Aldrich) in 95% ethanol (EtOH, Carlo Erba) and 1.22% glacial acetic acid (Carlo Erba). Sections were hydrated in EtOH solutions (100, 95, 70, and 50%), followed by staining with 0.1% LFB solution at 40°C for 40 min. Sections were then rinsed with tap water and differentiated in 0.05% Li_2_CO_3_ solution (Sigma–Aldrich). Sections were dehydrated in EtOH solutions (50, 70, 95, and 100%), cleared in xylene (Carlo Erba) and mounted with Entellan (Merck-Millipore) for light microscopy analysis of myelin content (Zeiss Axioscop 2).

### Myelin Content Quantification

After LFB staining, myelin content within the dorsal column of the spinal cords was quantified as percentage of the mean gray level within the dorsal column of each spinal cord slice (myelin positive pixels in the dorsal column/pixels of the total area of the dorsal column), using ImageJ software (U.S. National Institutes of Health). Blind quantification by three independent observers was performed to calculate the average value of the myelin content of healthy control, LPC-control and LPC-transplanted rats (*n* ≥ 20 slices/animal; *n* ≥ 3 animals/group).

### Statistical Analysis

As described for each methodology, *n* ≥ 3 animals or replicates were used for statistical analysis. Differences between experimental conditions were analyzed using two-way ANOVA followed by Tukey post-test. *P*-value < 0.05 was considered statistically significant.

## Results

### High Yield Oligodendrocytes from Rat Meningeal Biopsies: Development of a 4-Phases Oligodendrocyte Differentiation Protocol

**FIGURE 1:**
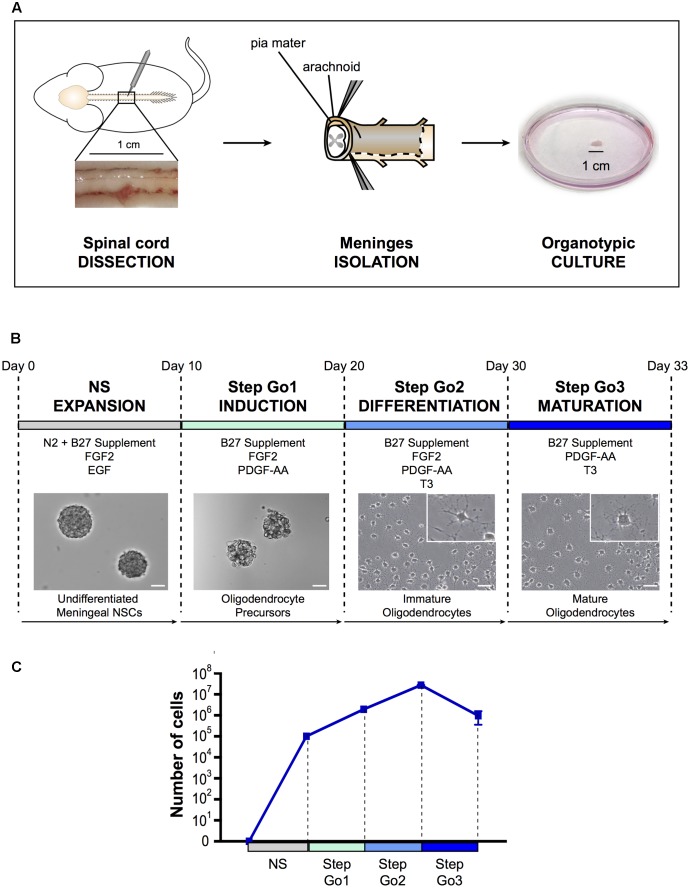
Oligodendrocyte differentiation protocol. **(A)** Schematic representation of spinal cord meningeal biopsy isolation for organotypic culture. Spinal cord was dissected from adult SD rat and 1 cm of meningeal tissue was isolated and plated in neurosphere expansion medium (NS, day 0). **(B)** Time course representation of the oligodendrocyte differentiation protocol from spinal cord meningeal biopsy. From day 0 to day 10: neurosphere expansion (NS), from day 10 to day 20: oligosphere culture (Step Go1); from day 20 to day 30: oligodendrocyte differentiation (Step Go2); from day 30 to day 33: oligodendrocyte maturation (Step Go3). Images show meningeal-derived differentiating oligodendrocyte morphology at each stage of the protocol. Insets in **(B)** are higher magnification images of representative cells in the boxes. Pictures in **(B)** are brightfield images. **(C)** Number of meningeal-derived cells in culture, calculated for every experimental replicate (*n* = 4), present at each stage of the differentiation protocol. Data are presented as mean ± SEM. NSCs: neural stem cells; FGF2: human basic fibroblast growth factor; EGF: epidermal growth factor; PDGF-AA: platelet-derived growth factor type AA; T3: 3,3′,5-triiodo-L-thyronine. Scale bars: 50 μm.

**FIGURE 2:**
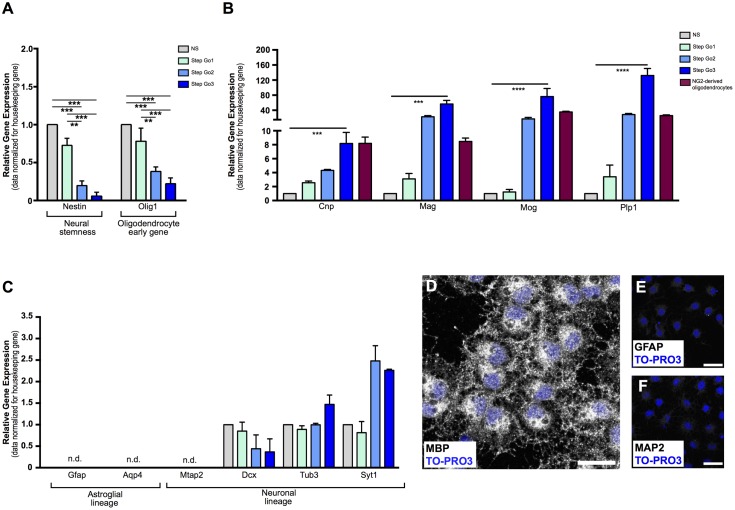
Gene and protein analysis confirms differentiation of meningeal-derived NSCs into oligodendrocytes. **(A)** Relative gene expression analysis of meningeal-derived oligodendrocyte lineage cells shows significant decrease of the neural-stemness-related gene *Nestin* and the oligodendrocyte-specification gene *Olig1* through the oligodendrocyte differentiation protocol. **(B)** Relative gene expression analysis of oligodendrocytes specific genes *Cnp*, *Mag*, *Mog*, and *Plp1* in meningeal-derived differentiating oligodendrocytes at each step of the differentiation protocol and in NG2-derived oligodendrocytes. As expected, Step Go3 meningeal-derived oligodendrocyte lineage cells show significant increase of oligodendrocyte specific genes compared to meningeal-derived cells in NS. **(C)** Gene expression analysis of specific astroglial lineage genes (*Gfap* and *Aqp4*) and neuronal lineage genes, (*Mtap2, Dcx, Tub3*, and *Syt1*) in meningeal-derived differentiation oligodendrocytes at each step of the differentiation protocol. *Gfap*, *Aqp4*, and *Mtap2* were not expressed at any step of the differentiation protocol, and were detected only after a high number of cycles (mean Δ*C*_t_: 17.2 ± 2.13 *Gfap*, 14.7 ± 1.66 *Aqp4*, and 13.89 ± 0.7 *Mtap2*). *Dcx*, *Tub3*, and *Syt1* were expressed at lower level during all steps of the differentiation protocol. Gene expression levels were normalized to those of the housekeeping gene β*-actin* and are expressed as normalized to basal conditions (NS). **(D–F)** Immunofluorescence analysis, showing that by the end of the protocol the majority of the meningeal-derived oligodendrocytes express the specific marker of mature oligodendrocyte MBP **(D)**, while none or rare cells express the specific astrocyte [GFAP, **(E)**] or neuronal [MAP2, **(F)**] markers. Data are presented as mean ± SEM; ^∗∗∗∗^*p* < 0.0001; ^∗∗∗^*p* < 0.001; ^∗∗^*p* < 0.01; ^∗^*p* < 0.05; n.d., not detectable. Images are single plane confocal images. Cell nuclei are visualized by TO-PRO3 nuclear staining (blue). Scale bars: 25 μm.

The possibility to perform *in vitro* patient-derived oligodendrocyte culture from adult somatic stem cells represents a potentially exploitable procedure for the identification of novel pharmacological targets for demyelinating disease and for high-throughput drug-screening assays (Franklin and Ffrench-Constant, 2008; Pino et al., 2017). Furthermore, autologous cell transplantation is the gold standard approach for cell therapy. Isolation of high numbers of mature oligodendrocytes from living adult CNS represents one of the main obstacles in cell transplantation translation to the clinic. Two major issues need to be addressed for cell therapy to be exploited as potential autologous cell transplantation: first, the location and size of the tissue to be sampled; second, the time needed to obtain a large number of transplantable cells. To overcome the lack of accessible adult sources for production of oligodendrocytes, we set up a protocol to obtain oligodendrocyte lineage cells from one single biopsy of adult rat superficial meningeal tissue. We optimized the protocol in order to simultaneously maximize meningeal-derived oligodendrocyte lineage cell expansion and differentiation. We divided the protocol into four phases that comprehend changing ratios of mitogens and differentiating morphogens, allowing both oligodendrocyte precursor expansion and gradual maturation into oligodendrocyte lineage cells (**Figure 1**). To assess the progressive enrichment of differentiating oligodendrocytes in culture, we analyzed, at each phase of the protocol, the gene expression of: NSC marker *Nestin* (**Figure 2A**) (Lendahl et al., 1990), oligodendrocyte precursor marker *Olig1* (**Figure 2A**) (Xin et al., 2005), and oligodendrocyte lineage markers coding for the major components of myelin sheaths *Cnp* (**Figure 2B**), myelin associated glycoprotein (*Mag*) (**Figure 2B**), myelin oligodendrocytes glycoprotein (*Mog*) (**Figure 2B**), and *Plp1* (**Figure 2B**) (Ranscht et al., 1982; Solly et al., 1996; Dugas et al., 2006; Cahoy et al., 2008).

#### NS – Neurosphere Induction

As for spinal cord central canal-derived NSCs, spinal cord meningeal-derived NSCs can be cultured *in vitro* and expanded as undifferentiated neurospheres (Weiss et al., 1996; Decimo et al., 2011). To obtain spinal cord meningeal-derived NSCs neurospheres, we sampled 1 cm^2^ biopsy of adult rat spinal cord meninges and cultured it directly, avoiding any mechanical-enzymatic procedure (**Figure 1A**). We used neurosphere-inducing medium supplemented with growth factors known to induce NSCs proliferation, such as EGF and FGF2 (Martens et al., 2000) (refer to section Media Compositions for media composition). In this phase, single cells shed form the meningeal biopsy and then grow as neurospheres in culture. After 7–10 days of culture, we observed floating neurospheres (**Figure 1B**), which consisted of cells expressing the NSC marker nestin (**Figure 2A**) (Lendahl et al., 1990) as SVZ-derived and spinal cord-derived NSCs (as described in Weiss et al., 1996; Decimo et al., 2011). In addition, meningeal-derived neurospheres expressed the early oligodendrocyte precursor marker *Olig1* (**Figure 2A**). We obtained approximately 2.5 × 10^5^ undifferentiated meningeal-derived NSCs after 10 days of culture (**Figure 1C**).

#### Step Go1–Oligodendrocyte Induction

To induce oligodendrocyte precursors differentiation and expansion, we continued to culture NS-dissociated cells in the presence of the mitogen FGF2 and of the oligodendrocyte inducing morphogen PDGF-AA (McKinnon et al., 1990; Engel and Wolswijk, 1996; Calver et al., 1998) for 10 days (**Figure 1B**). In this culture condition, cells continue to growth as spheres, now referred as “oligospheres” (**Figure 1B**). The proliferation rate increased compared to the neurosphere expansion phase (**Figure 1C**).

#### Step Go2 – Oligodendrocyte Differentiation and Proliferation

Subsequently, to induce immature oligodendrocytes differentiation from oligodendrocyte precursors, we changed culture conditions by adding the oligodendrocyte-differentiating hormone 3,3′,5-triiodo-L-thyronine (T3) (Almazan et al., 1985). To expand immature oligodendrocytes, we maintained in the medium the mitogens FGF2 and PDGF-AA, though PDGF-AA concentration was decreased (**Figure 1B**). Furthermore, to promote adhesion and extension of cellular processes typical of oligodendrocyte morphology (Baumann and Pham-Dinh, 2001), dissociated oligospheres were plated onto poly-D-lysine coated flasks and glass slides. After 7–10 days of culture in Step Go2 medium, we observed cells with branchings, suggesting a progression through oligodendrocyte lineage cells differentiation (**Figure 1B**). The immature oligodendrocytes statistically significantly decreased the expression levels of the stemness gene *Nestin* and of the oligodendrocyte precursor gene *Olig1* (**Figure 2A**), while the expression of mature oligodendrocyte markers *Cnp*, *Mag*, *Mog*, and *Plp1* (Ranscht et al., 1982; Campagnoni and Macklin, 1988; Cahoy et al., 2008) slightly increased (**Figure 2B**). In this culture condition, while inducing specific oligodendrocyte differentiation, we further promoted oligodendrocytes expansion and we were able to expand the cells ∼14-fold (**Figure 1C**).

#### Step Go3 – Oligodendrocyte Terminal Differentiation

To promote the final differentiation from immature oligodendrocytes to mature oligodendrocyte lineage cells, we removed the mitogen FGF2 from the medium, while maintaining the morphogens PDGF-AA and T3 (**Figure 1B**). After only 3 days of culture in Step Go3 medium, meningeal-derived oligodendrocyte lineage cells formed a dense network of fine processes typical of cultured mature oligodendrocytes (**Figure 1B**).

Gene expression analysis confirmed terminal oligodendrocyte differentiation, as shown by the upregulation of myelin-specific genes (Dugas et al., 2006) *Plp1*, (∼130-fold increase, *p* < 0.001 for Step Go3 vs. NS relative expression levels), as well as a 8-fold increase of *Cnp* (*p* < 0.001 compared to NS), 56-fold increase of *Mag* (*p* < 0.001 compared to NS) and 76-fold increase of *Mog* (*p* < 0.01 compared to NS) (**Figure 2B**).

To confirm that meningeal-derived oligodendrocyte lineage cells expressed the same oligodendrocyte markers of mature oligodendrocytes, we analyzed the oligodendrocyte marker expression of NG2-derived mature oligodendrocytes (Fumagalli et al., 2015) (**Figure 2B**, red bars). Meningeal-derived oligodendrocyte lineage cells (Step Go3) and NG2-derived oligodendrocytes expressed comparable levels of *Cnp*, *Mog*, *Mag*, and *Plp1* specific oligodendrocyte genes (**Figure 2B**).

Altogether, these results indicate that the protocol allows a gradual *in vitro* differentiation of meningeal-derived NSCs toward the mature oligodendroglial lineage. Indeed, meningeal-derived NSCs progressively increase the specific oligodendrocyte-related gene expression levels while downregulating immature NSC genes.

### Meningeal-Derived NSCs Differentiated Homogeneously into Oligodendrocyte Lineage Cells

**FIGURE 3:**
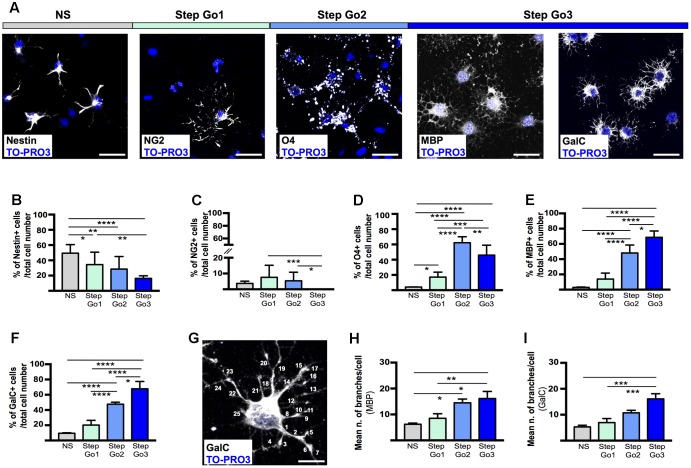
Stage-specific oligodendrocyte differentiating marker expression. **(A)** Representative immunostaining images of meningeal-derived differentiating oligodendrocytes (white) at each stage of the differentiation protocol. In NS stage, meningeal-derived cells express the specific marker of stemness, nestin; in Step Go1, meningeal-derived cells express the specific marker of oligodendrocyte precursors, NG2; in Step Go2, meningeal-derived cells express the specific marker of immature oligodendrocytes, O4 and finally in Step Go3 meningeal-derived cells express specific markers of mature oligodendrocytes, MBP and GalC. **(B–F)** Graphs representing the percentage number of nestin^+^ **(B)**, NG2^+^ **(C)**, O4^+^ **(D)**, MBP^+^ **(E)**, and GalC^+^ **(F)** cells among the total counted cells at each stage of the oligodendrocyte differentiation protocol. In **(B)**, nestin^+^ cells significantly decrease along the oligodendrocyte differentiation protocol (NS vs. Step Go1, NS vs. Step Go2, NS vs. Step Go3). In **(C)**, NG2^+^ cells, increase from NS to Step Go1 and decrease in the following steps. In **(D)**, O4^+^ cells peak at Step Go2 (Step Go2 vs. NS and Step Go2 vs. Step Go1). In **(E,F)**, MBP^+^ and GalC^+^ cells significantly increase in Step Go3. **(G–I)** Representative immunostaining image **(G)** and quantification graphs of the mean number of cellular branches per cells **(H,I)** in Step Go3 cells, stained with specific markers for mature oligodendrocytes, MBP **(H)**, and GalC **(G,I)**. These data show that the number of branches significantly increases along the oligodendrocyte differentiation protocol and highlight the maturation of meningeal-derived oligodendrocytes obtained at Step Go3 (MBP: Step Go2 vs. NS, Step Go3 vs. NS, Step Go3 vs. Step Go1; GalC: Step Go3 vs. NS, Step Go3 vs. Step Go1). Quantitative data are mean ± SEM; ^∗∗∗∗^*p* < 0.0001; ^∗∗∗^*p* < 0.001; ^∗∗^*p* < 0.01; ^∗^*p* < 0.05. All the images single plane confocal images. Cell nuclei are visualized by TO-PRO3 nuclear staining (blue). Scale bars: 25 μm.

Cell transplantation, as well as drug screening assay, requires high numbers of pure, homogeneously differentiated mature cells. We therefore tested whether our protocol induced differentiation of meningeal-derived NSCs specifically into mature oligodendrocytes, with no contamination of other cell types. To assess the purity of the differentiated meningeal-derived oligodendrocyte lineage cell population, we analyzed the gene expression of neuronal and glial genes during all phases of the differentiation protocol. We could not detect expression of the neuronal-specific gene *Mtap2* (Izant and McIntosh, 1980) and of the astrocyte-specific genes *Gfap* (Eng, 1985) and *Aqp4* (Yoneda et al., 2001) (**Figure 2C**) in each phase of the protocol analyzed. In line with previous reports (Cahoy et al., 2008), we detected low levels of expression of the neural precursor marker *Dcx* (Brown et al., 2003) the immature neuronal marker class III β-tubulin (*Tub3*) and the synaptic protein synaptotagmin 1 (*Syt1*) (**Figure 2C**). In accordance to previous findings (Cahoy et al., 2008), although low in absolute values, *Tub3* and *Syt1* increased approximately two-fold in the last oligodendrocyte differentiation phase (Step Go3) (**Figure 2C**). As confirmation, immunofluorescence analysis revealed that by the end of the protocol, the majority of the cells expressed the oligodendrocyte specific protein MBP (**Figures 2D**, **3A,E**), while none or rare cells were positive for the astrocyte marker GFAP (Eng, 1985) and the neuronal marker MAP2 (Izant and McIntosh, 1980) (**Figures 2E,F**).

These data indicate that the meningeal-derived oligodendrocyte lineage cell culture does not include cells belonging to astrocyte or neuronal lineages, suggesting that they homogenously differentiated into oligodendrocytes.

### Evaluation of Meningeal-Derived Oligodendrocyte Lineage Cell Maturation

To further assess the degree of maturation of the meningeal-derived oligodendrocyte lineage cells at each phase of the protocol, we assessed and quantified by immunofluorescence analysis the number of differentiating cells expressing the NSC marker nestin (Lendahl et al., 1990) (**Figures 3A,B**), the oligodendrocyte precursor marker NG2 (**Figures 3A,C**), the immature/intermediate oligodendrocyte progenitor marker O4 (Gard and Pfeiffer, 1989) (**Figures 3A,D**) the myelin component MBP (Campagnoni and Macklin, 1988) and GalC (Ranscht et al., 1982) (**Figures 3A,E,F**). We found that the majority of the neurospheres (NS) expressed, as expected, the NSC marker nestin (**Figures 3A,B**). Following oligodendrocyte precursor induction (Step Go1), nestin expression was decreased, while the early oligodendrocyte precursor marker (NG2) was statistically increased (**Figures 3A–C**). At this stage, the intermediate and mature oligodendrocyte markers were expressed at low levels (**Figures 3C–F**). In Step Go2, the immature oligodendrocyte marker O4 was statistically increased, while nestin and the early oligodendrocyte marker NG2 expression was decreased, suggesting that meningeal-derived NSCs were progressively differentiating into immature oligodendrocyte lineage cells (**Figures 3A–D**). The myelin components GalC and MBP, typically expressed by mature oligodendrocytes (Campagnoni and Macklin, 1988) were slightly increased in Step Go2 and were statistically significantly increased in Step Go3, indicating that meningeal-derived oligodendrocyte lineage cells have reached the maximum stage of the maturation process by the end of the differentiation protocol (**Figures 3A,E,F**).

Mature oligodendrocytes extend numerous processes; thus, to evaluate the degree of differentiation of the meningeal-derived oligodendrocyte lineage cells we quantified the number of cell branchings (Pfeiffer et al., 1993). To quantify the branchings, we immunostained for MBP and GalC the differentiating oligodendrocyte and we counted the branchings extending from each single cell through every step of the protocol (**Figures 3G–I**). We found that the number of branchings for each cell progressively statistically increased by reaching a mean of about 20 branchings/cell, typical of mature oligodendrocytes (Butt et al., 1994) in the last phase of the protocol (**Figures 3G–I**).

Altogether, those data suggest the meningeal-derived neurospheres are induced to differentiate progressively to oligodendrocyte precursors, immature oligodendrocytes and mature oligodendrocyte lineage cells.

### *In Vivo*-Remyelinating Potential of Meningeal-Derived Oligodendrocytes

**FIGURE 4:**
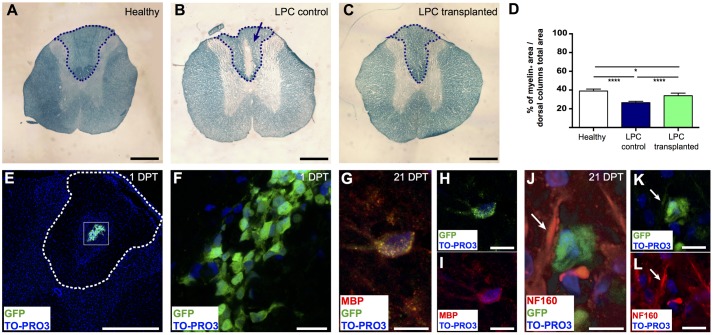
Remyelinating potential of transplanted meningeal-derived oligodendrocytes. **(A–C)** Brightfield images of spinal cord transversal sections of healthy, LPC-control, and LPC-transplanted rats stained with specific myelin staining LFB. LFB allows the identification of the myelin content of the tissue (blue) from the demyelinated area (white). While spinal cord sections of healthy rat did not show any evidence of demyelination **(A)**, spinal cord sections of LPC-control rats showed a focal demyelination in the injection sites, as indicated by the blue arrow **(B)**. **(C)** Spinal cord sections of LPC-transplanted rats showed an higher intensity of the LFB staining around the injection site compared to LPC-control rat sections. **(D)** The graph represents the percentage of the myelin content in the dorsal column of the spinal cord sections of healthy (38.94% ± 1.2%), LPC-control (26.42% ± 0.6%), and LPC-transplanted (33.97% ± 1.1%) rats, calculated as myelin positive pixels in the dorsal column among the pixels of the total area of the dorsal column. The blue dashed lines in **(A–C)** indicate the dorsal column areas of the spinal cord sections of healthy, LPC-control, and LPC-transplanted rats, that represent the LPC-lesioned area considered for the myelin content quantification. The analysis shows that transplantation of meningeal-derived oligodendrocytes resulted in a significantly increased myelin content percentage in LPC-transplanted sections compared to LPC-controls sections. Quantitative data are expressed as means ± SEM; *n* = 3 in healthy animals, *n* = 6 in LPC animals; ^∗^*p* ≤ 0.05, ^∗∗∗∗^*p* ≤ 0.0001. The average value was calculated for healthy control, LPC-control and LPC-transplanted rats (*n* ≥ 20 slices/animal). **(E,F)** Immunostaining for GFP (green) and TO-PRO3 nuclei (blue) in a spinal cord section of a LPC-transplanted rat at 1 DPT, showing that eGFP^+^ meningeal-derived oligodendrocytes were localized inside the spinal cord parenchyma close to the LPC lesion site. The white dashed line in **(E)** indicates the dorsal columns of the spinal cord, **(F)** is a higher magnification of the box in **(E)**. **(G-I)** Immunostaining of a spinal cord section of a LPC-transplanted rat at 21 DPT, showing that eGFP^+^ meningeal-derived oligodendrocytes (green) co-express the specific marker for mature oligodendrocytes, MBP (red). Merged image in **(G)**; GFP (green) and TO-PRO3 (blue) in **(H)**; MBP (red) and TO-PRO3 (blue) in **(I)**. **(J–L)** Immunostaining of a spinal cord section of a LPC-transplanted rat at 21 DPT, showing that eGFP^+^ meningeal-derived oligodendrocytes (green) are in close contact to the neuron neurofilament, stained with NF160 (white arrows in **(J–L)**. Merged image in **(J)**; GFP (green) and TO-PRO3 (blue) in **(K)**; NF160 (red) and TO-PRO3 (blue) in **(L)**. **(E)** and **(F)** are maximum Z-projection images of confocal images. Scale bars: 1 mm **(A–C)**, 500 μm **(E)**, 20 μm **(F,H,I,K,L)**, 40 μm **(G,J)**.

To assess the *in vivo* remyelinating potential of the meningeal-derived oligodendrocyte lineage cells, we developed a controlled model of *in vivo* focal spinal cord chemical demyelination by injecting the demyelinating drug LPC in the dorsal columns of the spinal cord. After 7 days from the injection of LPC (7 DPI), a focal demyelinated area was clearly evident at the dorsal column region of the spinal cord parenchyma. We transplanted eGFP^+^ meningeal-derived oligodendrocytes into the demyelinated area at 7 DPI and we analyzed their myelinating potential 21 days after the transplantation (21 DPT) [see Materials and Methods section Quantitative Real-time RT (Reverse Transcription)-PCR Analysis (qRT-PCR)]. LFB staining of the spinal cords of healthy control (not injured), LPC-control (injected with vehicle) and LPC-transplanted animal group at 21 DPT showed the difference in the myelin content among the groups (**Figures 4A–C**). Importantly, myelin quantification in the dorsal column region of the spinal cords showed a statistical significant increase of the percentage of myelin in the spinal cords of LPC-transplanted group compared to the LPC-control group (**Figure 4D**).

To confirm the presence of transplanted eGFP^+^ meningeal-derived oligodendrocyte lineage cells in LPC-transplanted rats, we analyzed their location and fate. As expected, at 1 DPT immunofluorescence analysis revealed that eGFP^+^ meningeal-derived oligodendrocyte lineage cells were localized in the spinal cord parenchyma (**Figures 4E,F**) of all LPC-transplanted animals. Healthy and LPC-control rats did not exhibit such labeling. At 21 DPT, eGFP^+^ cells persist in the LPC-lesion region and expressed MBP (**Figures 4G–I**). The immunostaining for neurofilament (NF160) suggested that eGFP^+^ transplanted cells were in close contact with axons that maintained their integrity after the treatment with LPC (**Figures 4J–L**).

## Discussion

In this study, we described an efficient method to obtain high yield of oligodendrocyte lineage cells from a small rat meningeal biopsy. Our aim was to develop a protocol potentially applicable for cell therapy and for *in vitro* drug screening.

Although significant progresses have been made in developing pharmacological therapies to increase oligodendrocyte lineage cell number and optimize their differentiation, there is still an unmet need for translating successful remyelination in clinical setting. *In vitro* oligodendrocyte cultures can be useful for both cell therapy and drug screening purposes. Currently, *in vitro* expansion of oligodendrocyte lineage cells can be obtained by (i) sorting of oligodendrocyte precursors from postnatal or adult brain tissue (Zhu et al., 2007; Pedraza et al., 2008, 2014; Fumagalli et al., 2011, 2015; Dugas and Emery, 2013a,b; Emery and Dugas, 2013; Medina-Rodríguez et al., 2013; Lu et al., 2015); (ii) culturing and differentiating ESCs into oligodendrocytes (Glaser et al., 2004; Zhang et al., 2004; Chojnacki and Weiss, 2008; Jiang et al., 2010; Neri et al., 2010; Sundberg et al., 2010; Sharp et al., 2011; Neman and de Vellis, 2012; Alsanie et al., 2013; Franco et al., 2015; Kerman et al., 2015; Wang et al., 2015; Yamashita et al., 2017; Yao et al., 2017) and (iii) generating and differentiating oligodendrocytes from patient-derived iPSCs (Khazaei et al., 2007; Hu et al., 2009; Czepiel et al., 2011; Ogawa et al., 2011; Sundberg et al., 2011; Douvaras and Fossati, 2015; Gorris et al., 2015; Li et al., 2016; Kim et al., 2017; Rodrigues et al., 2017). All these available methods present some pitfalls that limit their clinical exploitation. *In vitro* culture of sorted oligodendrocyte precursors requires sampling of sizable brain tissue and does not always provide a pure oligodendrocyte expansion. ESCs have remarkable long-term proliferative potential, providing the possibility of unlimited expansion in culture and a broad differentiation potential. However, there are important ethical and safety issues, including the need of immunosuppressant therapy that increases the risk of teratoma formation. The production of oligodendrocyte-like cells directly from induced patient somatic cells is the most promising technique for autologous transplantation purposes. The risk of tumorigenicity, however, dampens its clinical applicability (Ben-David and Benvenisty, 2011). Transplantation of high numbers of autologous mature oligodendrocytes from living adult subject would represent the gold standard approach for cell therapy.

We identified in meninges the presence of NSCs, endowed of neural differentiation potential both *in vitro* and *in vivo* (Bifari et al., 2009, 2015, 2017; Decimo et al., 2011, 2012a,b). Meninges are a more accessible tissue compared to brain and spinal cord parenchyma. Moreover, NSCs are retained in adult brain and spinal cord meninges, thus there is no need of artificial *in vitro* transformation. We therefore developed a protocol to obtain oligodendrocyte lineage cells derived from adult rat meningeal biopsy. We optimized the protocol to address the most relevant issues for clinical translation including (i) short time of *in vitro* cell expansion, (ii) well-defined media conditions, and (iii) homogeneous phenotype of differentiated cells. We obtained a high number of meningeal-derived oligodendrocyte lineage cells (10 million cells) in a relatively short period of time (approximately 4 weeks). In addition, *in vitro* meningeal-derived oligodendrocyte lineage cell expansion and differentiation were carried out in well-defined culture media (in the absence of serum) and adhesion substrate (poly-D-lysine), therefore enhancing standardization and the potential clinical translation of the protocol. We cultured the whole rat meningeal biopsy avoiding intermediate steps of enzymatic and/or mechanical dissociation, in order to minimize *in vitro* manipulation and maximizing cellular viability. Meningeal-derived NSCs were differentiated into a homogeneous culture of mature oligodendrocyte lineage cells as suggested by the expression of oligodendrocyte markers (GalC and MBP) and the lack of expression of neuronal and astrocyte markers (Map2 and GFAP) (**Figures 2E,F**) (Campagnoni and Macklin, 1988; Butt et al., 1994). Indeed, meningeal-derived oligodendrocyte lineage cells express comparable levels of oligodendrocyte specific genes to those of mature NG2-derived oligodendrocytes (**Figure 2B**) (Cahoy et al., 2008).

Notably, meningeal-derived oligodendrocyte lineage cells showed *in vivo* remyelinating potential (Liu et al., 2000; Razavi et al., 2017). To assess the *in vivo* differentiation and remyelinating potential of the meningeal-derived oligodendrocyte lineage cells, we used the animal model of focal spinal cord chemical demyelination, by injecting the drug LPC. Our results indicate that meningeal-derived oligodendrocyte lineage cells are endowed of *in vivo* differentiation and remyelinating potential. Although LPC-induced demyelination is a valuable tool for screening candidates for remyelination-promoting therapies, this model did not include all the complex interactions (i.e., vascular and autoimmune) occurring in the most common demyelinating diseases such as stroke and MS. In this work, we aimed to first assess the *in vivo* remyelinating potential rather than the overall therapeutic effect of the meningeal-derived oligodendrocyte linage cells. We therefore choose the animal model of chemical LPC demyelination. Previous works suggest that meningeal resident NSCs react to brain and spinal cord damage (Decimo et al., 2011; Nakagomi et al., 2011, 2012; Ninomiya et al., 2013) by increasing their stemness and differentiation potential. However, whether meningeal resident NSCs react similarly following complex demyelinating diseases remains to be determined.

The development of a successful protocol for OPC/oligodendrocyte lineage cell culture of adult origin could provide a useful tool for the *in vitro* screening and testing of drugs able to influence the biology and remyelinating potential of OPCs (Allen et al., 2005; Soldatow et al., 2013; Kerman et al., 2015). Different strategies are now under investigation for *in vitro* drug screening, as the use of primary cultures, including OPCs (Merrill, 2008; Gonzalez et al., 2016; Lariosa-Willingham et al., 2016), iPSCs (Iwata et al., 2017; Rana et al., 2017), organoids (Vrij et al., 2016; Pino et al., 2017), spheroids (Sarkar et al., 2017; Sirenko et al., 2017), and bioprinted 3D tissues (Chang et al., 2010; Massa et al., 2017). However, none of these methods is able to provide adult patient-specific oligodendrocytes without major *in vitro* transformation. On the contrary, meningeal-derived oligodendrocyte lineage cells may be potentially used for precision medicine to develop a patient-specific assay to test drugs, starting from a population of meningeal cells extracted directly from the living patient.

Overall, our protocol has potential of translation and application in autologous setting. Since, we harvested meninges covering the spinal cord, we avoided dangerous invasive sampling of the delicate CNS tissue. Moreover, we developed a protocol to produce a sufficient number (>10 millions) of transplantable cells starting from a single donor tissue extraction. Therefore, this method may be applicable in autologous settings, as a small meningeal biopsy could potentially be harvested from a subject and directly cultured *in vitro* to obtain high yield of transplantable meningeal-derived oligodendrocyte lineage cells. Subsequently, *in vitro* expanded and differentiated meningeal-derived oligodendrocyte lineage cells could be transplanted in the same donor from which the meninges were sampled. This protocol may be exploited in the future to obtain oligodendrocytes for cell therapy of different demyelinating disease models, including MS, stroke and traumatic brain and spinal cord injuries, thus further testing the therapeutic potential of meningeal-derived oligodendrocyte lineage cells.

## Conclusion

The physiological function of adult meningeal-resident NSCs, as well as their complete cellular and molecular characterization, is only partially known. The presence of meningeal-resident progenitor cells has, however, been reported both in adult rodent and humans (DeGiorgio et al., 1994; Bifari et al., 2009, 2015, 2017; Decimo et al., 2011, 2012a,b; Petricevic et al., 2011). Meningeal-resident NSCs have been shown to react to CNS damage (Decimo et al., 2011; Nakagomi et al., 2011, 2012; Ninomiya et al., 2013). With this work, we identified meninges as an optimal source of adult NSCs, that can be easily isolated, expanded, and differentiated into oligodendrocyte lineage cells. These cells express the phenotypic and genetic markers of *bona fide* oligodendrocytes, are functional and able to restore myelin content in a chemical demyelinating model. However, how these *in vitro* generated meningeal-derived oligodendrocyte lineage cells may survive and what is their regenerative potential in different demyelinating pathological microenvironment, such as MS, ischemic, and traumatic injuries, will need further investigations.

## Author Contributions

SD, AP, VB, FB, and ID designed the study, performed the experiments, and analyzed and interpreted data. FP, AB, SZ, CA, DM, GM, and MF assisted with molecular analyses, animal work, and histochemical analysis. PG and FJR set-up the LPC spinal cord lesion and transplanted the cells. GM performed gene expression analysis. MF and EB cultured and differentiated NG2-derived oligodendrocytes. SD, AP, VB, GF, FB, and ID wrote the paper. All authors discussed results and commented on the manuscript. FB and ID conceptualized the study, supervised the project, and have the scientific direction.

## Conflict of Interest Statement

The authors declare that the research was conducted in the absence of any commercial or financial relationships that could be construed as a potential conflict of interest.

## Abbreviations

Aqp4: aquaporin 4
Cnp: 2′,3′-cyclic-nucleotide 3′-phosphodiesterase
CNS: central nervous system
Dcx: doublecortin
EGF: epidermal growth factor
ESCs: embryonic stem cells
FGF2: human basic fibroblast growth factor
GalC: galactosylceramidase
GFAP: glial fibrillary acidic protein
iPSCs: induced-pluripotent stem cells
LFB: Luxol Fast Blue
LPC: lysophosphatidylcholine
MAP2/Mtap2: microtubule-associated protein 2
MBP/Mbp: myelin basic protein
MS: multiple sclerosis
NF160: neurofilament 160
NG2: chondroitin sulfate proteoglycan
NSCs: neural stem cells
OECs: olfactory ensheathing cells
OPCs: oligodendrocyte precursor cells
PDGF-AA: platelet-derived growth factor-AA
PDGFRα: platelet-derived growth factor receptor type α
Plp1: proteolipid protein 1
SVZ: subventricular zone
Syt1: synaptotagmin 1
T3: 3,3′,5-triiodo-L-thyronine
Tub3: class III β tubulin
